# Automatic detection of problem-gambling signs from online texts using large language models

**DOI:** 10.1371/journal.pdig.0000605

**Published:** 2024-09-25

**Authors:** Elke Smith, Jan Peters, Nils Reiter

**Affiliations:** 1 Department of Psychology, Biological Psychology, University of Cologne, Germany; 2 Department of Digital Humanities, University of Cologne, Germany; Intel Corporation, UNITED STATES OF AMERICA

## Abstract

Problem gambling is a major public health concern and is associated with profound psychological distress and economic problems. There are numerous gambling communities on the internet where users exchange information about games, gambling tactics, as well as gambling-related problems. Individuals exhibiting higher levels of problem gambling engage more in such communities. Online gambling communities may provide insights into problem-gambling behaviour. Using data scraped from a major German gambling discussion board, we fine-tuned a large language model, specifically a Bidirectional Encoder Representations from Transformers (BERT) model, to predict signs of problem-gambling from forum posts. Training data were generated by manual annotation and by taking into account diagnostic criteria and gambling-related cognitive distortions. Using cross-validation, our models achieved a precision of 0.95 and F1 score of 0.71, demonstrating that satisfactory classification performance can be achieved by generating high-quality training material through manual annotation based on diagnostic criteria. The current study confirms that a BERT-based model can be reliably used on small data sets and to detect signatures of problem gambling in online communication data. Such computational approaches may have potential for the detection of changes in problem-gambling prevalence among online users.

## 1 Introduction

Gambling refers to the act of betting or wagering on an event with an uncertain outcome, with the primary intent of winning money or material goods. This activity is usually associated with games of chance, such as slot machines, lotteries, and card games. In contrast to gaming, which contains elements of strategy or competition, the gambling outcomes are primarily determined by chance. In some individuals, however, gambling behaviour can escalate and result in a loss of control over gambling behaviour. Disordered gambling can then lead to severe psycho-social consequences. Affected individuals typically feel an urge to gamble and are preoccupied with gambling, often have difficulties to control or stop gambling, gamble high amounts of money and try to compensate for losses by further gambling [1]. Disordered gambling commonly leads to psychosocial distress, financial problems, problems at work and in the family, and heightened levels of stress [2]. In addition to escalating gambling behaviour and resulting problems such as financial difficulties, disordered gambling is linked to specific gambling-related cognitive distortions, i.e. irrational and superstituous cognitions. For example, individuals overestimate their chances of winning, downplay the risks associated with gambling and hold maladaptive and erroneous beliefs about the role of skill in gambling (illusion of control) [3, 4].

Disordered gambling is a mental disorder listed in the Diagnostic and Statistical Manual of Mental Disorders (5th ed., DSM-5) [1], and referred to as pathological gambling in the International Classification of Diseases (11th revision, ICD-11) [5]. Prevalence rates for disordered gambling vary from country to country, but figures between 0.1 and 0.6 percent are reported for Europe [6]. The condition is strongly comorbid with a range of psychiatric symptoms, including anxiety, depression and substance use [7], making it a major public health concern [8].

In Germany, land-based (terrestrial) gambling is subject to several regulations and restrictions such as mandatory programmes for self-exclusion, age and marketing restrictions [9, 10]. Following a revision of the German State Treaty on Gambling in 2021 (GlüStV 2021), online gambling was legalised in 2021 [10]. Gambling behaviour and gambling-associated problems are often identified through self-report, including interviews and questionnaires. These, in turn, are subject to known biases such as social desirability [11] and recall bias [12], which describe the tendency of respondents to give answers that are assumed to be viewed as favourable by others, and the inaccurate recall of past events, respectively. Thus, more direct measures of behaviour might be more informative in some cases. However, a direct assessment of gambling behaviour is technically challenging and would require online tracking or on-site observations and provider cooperation.

### 1.1 Problem gambling and participation in online communities

On the internet, there are numerous gambling communities and online discussion boards where users exchange information on gambling experiences, putative strategies and gambling-related problems [13, 14]. Survey studies report that individuals who exhibit higher levels of problem gambling engage more in such communities [15, 16]. For instance, in a Finnish survey, more than half of online gambling forum users reported some problems with gambling [15], as measured by the South Oaks Gambling Screen (SOGS) [17]. This highlights the importance of understanding the experiences and problems related to gambling that are shared in these online forums. The study of online gambling communities may therefore be a fruitful way to provide further insights into gambling behaviour and associated problems [13]. Since research suggests a role of online communities in the development and persistence of problem gambling, monitoring online gambling communities could further be useful in initiating low-threshold preventive measures [15, 16]. There are both qualitative and quantitative approaches, and combinations of both, to study content from online communities [13, 18, 19]. Given the large amounts of text-based information contained in these communities, computational methods for information extraction have been widely adopted, for instance, to predict mental health conditions by using social media posts [20, 21].

### 1.2 Computational linguistics in clinical psychology

To derive information with potential clinical or public health implications from large bodies of text, e.g. with respect to clinical psychological questions, computational linguistics approaches can be helpful. The application of natural language processing (NLP) methods in clinical settings may facilitate dealing with big data, for instance by retrieving infomation with potential clinical or public health implications, and by structuring and synthesising information from clinical documents and scientific literature [22, 23]. For instance, performing computerised lexical analysis of narratives from individuals with gambling or buying compulsions with software for Linguistic Inquiry and Word Count (LIWC) [24] revealed differences in emotional tone and authenticity between compulsive buying and gambling narratives, suggesting that differences in phenomenology may be automatically identified from written narratives.

### 1.3 Automatically predicting signs of problem gambling

Automatically detecting problem-gambling content, indicating potentially problematic gambling behaviour and early signs of gambling addiction from online texts could aid website operators in their monitoring activities in the context of player protection. Such approaches may also provide researchers with tools for measuring changes in online posting behaviour that might be related to specific events such as lockdowns following the COVID-19 pandemic in 2020, or the introduction of the GlüStV 2021 in Germany [10]. To date, few studies have specifically addressed the issue of automatically detecting signs of problem gambling from online posts.

There is some published work from the 2021 and 2022 editions of the Early Risk Detection on the Internet (eRisk) workshop [25, 26]. The eRisk workshop contributes to developing and evaluating computational methods to detect risk factors for mental health problems on the internet, including depression and problem gambling. Relying on texts from Reddit, and following a user-centred approach, users who had posted or commented in subreddits dealing with problem gambling or gambling addiction were considered as users at-risk, while control users were either taken from the publicly available Reddit Self-reported Depression Diagnosis (RSDD) [27] and eRisk 2018 depression [28] datasets, or obtained by selecting users which had posted in gambling-unrelated subreddits.

Evaluating machine learning classification performance is typically based on precision, recall, and F1 score. Precision denotes the fraction of target class elements among the retrieved elements, while recall (also referred to as sensitivity) denotes the fraction of target class elements that were retrieved. The F1 score is a combined measure of precision and recall, specifically the harmonic mean between the two. The metrics range between 0 and 1, with higher scores indicating better classification performance. Training different machine learning models at eRisk [25, 26] for predicting early signs of problem gambling, yielded F1 scores of 0.72 [29] and 0.87 [30], when using a BoW representation with a support vector machine (SVM), and approximate nearest neighbour algorithm, respectively. Also, Bucur and colleagues [31, 32] fine-tuned a pre-trained Bidirectional Encoder Representations From Transformers (BERT) model to the task of predicting signs of problem gambling from gambling-related Reddit posts, yielding F1 scores of 0.27 and 0.41, respectively. While recall was high (0.98 and 0.99), precision was rather low (0.16 and 0.26).

The aforementioned works demonstrate the feasibility of using machine learning algorithms and models, such as SVM and BERT, for automatically predicting signs of problem gambling. However, model performance was in some cases limited, thus leaving room for improvement, especially with regard to precision. Considering the potential applications of such models, e.g. for early detection of at-risk behaviours from social media, obtaining high precision is an essential requirement to avoid producing massive amounts of false positives. One potential reason for the low precision in previous work concerns the way in which the training material was constructed. The training data were not manually annotated, rather, all posts from the chosen subreddits were defined as positive class items, assuming that the majority of users posting there exhibit pathological gambling behaviour. In addition to concerns regarding validity, this may have led to significant numbers of posts from non-problem-gambling users being included in the positive (at-risk for problem gambling) class.

Building upon this, the aim of the present work was to automatically identify problem-gambling content in online forum posts using a pre-trained ML model for NLP. We expanded upon previous work by using manual annotation to define training labels. Annotation was based on both diagnostic criteria and gambling-related cognitions. Regarding diagnostic criteria, descriptions of one’s gambling behaviour in forum posts may provide information about whether an individual describes problems with gambling, such as gambling-related financial problems, urges to gamble or issues related to treatment and support. Regarding gambling-related cognitions, posts discussing gambling strategies or tactics might contain signatures of maladaptive beliefs, for instance listing lucky numbers. To this end, posts scraped from a large German online gambling forum were manually annotated as containing problem-gambling content vs. containing gambling content only, based on standard diagnostic instruments for problem gambling. We then fine-tuning a pre-trained German version of the BERT model [33] using the annotated forum posts to examine the degree to which such content types could be automatically detected.

## 2 Materials and methods

### 2.1 Data collection and description

The data used in the current work stem from a discussion board that is part of a German-speaking online casino and gambling website. The topics discussed centre around online casinos, slot machines, games such as roulette, blackjack and poker, and gambling addiction. The website was scraped using Python (version 3.6.9) [34], and the Requests (version 2.18.4) and Beautiful Soup (version 4.6.0) [35] libraries. The data scraped from the website were openly accessible and technical measures designed to prevent web scraping were not disregarded. The scraped XML data were parsed into a relational SQLite database [36] using the sqlite3 module for Python. The database contains a total of 205,385 forum posts, as well as metainformation about posts, including publication date and URL (specifying the respective subforum). The gambling addiction subforum contains 4,150 posts, 202 of which are initial posts. All other forum sections (excluding the gambling addiction subforum) contain 201,235 posts, including 7,705 initial posts (i.e., posts that are not replies to another post). The discussion board is grouped into eleven superordinate board topics (see Table 1), with the highest number of posts found in the online casino subforum. Text length (tokens per post) is, on average, twice as high in the gambling addiction subforum (*M* = 296.16) compared to all other subforums (*M* = 141.54).

**Table 1:** Distribution of posts per subforum.

Subforum	*N* posts
Rules and guidelines	9349
Blackjack	308
Poker	296
Roulette	614
Other games of chance	4701
Slot machines and slot games	6079
Gambling arcades and casinos	3365
Casino complaints	14328
Gambling addiction	4150
Online casinos	140818
Miscellaneous	21377

### 2.2 Ethical considerations

For independent scientific research that uses web scraping of publically available data, no consent is required under the following conditions: The information to be evaluated must be generally accessible; technical measures designed to prevent web scraping may not be disregarded; the research may only serve non-commercial purposes; the use of web scraping technologies must not cause technical damage to the operator of the website. The transmission of the scanned corpus to scientific journals is not permitted [37]. For the current analysis, only forum posts were processed, such that the processed data did not contain personal data, such as information about individual user profiles or any other data about individual users. Since we do not know the users’ identities, consent was not obtained. This is in line with a range of previous studies processing publically accessible user posts from Reddit and Twitter [31, 38, 39]. The ethical justification for scraping publicly available forum posts from a discussion board lies in the context of the data’s accessibility and the nature of the information shared [37, 40]. In the present case, the scraped discussion board is clearly recognisable as publicly visible, and no registration is required to view posts. It can therefore be assumed that individuals contributing to the discussion board implicitly understand that their posts become part of a public forum and are accessible to other members and the broader public.

### 2.3 Selection of gambling forum posts

Since BERT has a maximum length limit of 512 tokens (see also section 2.6), only posts with length ≤ 512 tokens were considered for annotation. Using all posts and automatically truncating them before classification would result in the annotated data not being comparable to the training data. It is conceivable that a post contains descriptions after the 512th token that make it a target. Only initial posts (i.e., posts that are not replies to other posts) were considered for annotation. After removing all posts with a token length > 512, 168 initial posts remained from the addiction subforum, and 7466 initial posts from all other subforums. The gambling addiction subforum potentially contains more target (i.e. problem gambling) posts, whereas posts from all other subforums potentially contain more non-target (i.e. gambling only) posts. Since the gambling addiction subforum contains few initial posts, all posts from this forum were exported for manual annotation (*N* = 168). Twice as many posts were exported from all other subforums (*N* = 336, randomly selected). The number of posts used for annotation are listed in Table 2.

**Table 2:** Overview on the posts used for annotation and training.

	*N* per subforum	
	Misc^a^	GA
Initial and reply posts	201,235	4,150
Initial posts	7,705	202
Initial posts with length ≤ 512	746 6	168
Posts used for annotation	336	168

		*N* assigned class labels		
		G	PG	None^b^
	Total	348	138	18
Training sets	1/2 of PG posts	69	69	-
	2/3 of PG posts	92	92	-
	All PG and G posts	348	138	-
	All PG and G posts plus upsampled PG posts	348	348	-

^a^ All subforums except for the gambling addiction subforum

^b^ Inconclusive, empty, deleted or advertisement posts

*Note*. GA: gambling addiction; G: gambling; PG: problem gambling.

Text length (in tokens, as determined through tokenisation with the NLTK Tokenizer package [41]) was, on average, higher in the gambling addiction subforum compared to all other subforums (see section 2.1), even after removing posts with token length > 512. To prevent the classifier from using text length as a criterion, exporting posts from all non-gambling addiction subforums was performed with weighted random sampling based on text length to obtain similar distributions in terms of token length. The difference in token length between posts exported from the addiction subforum and the random weighted sample of posts from all other subforums was not significantly different (*T* = -0.99, *p* = 0.32, Welch’s t-test). To prevent the annotation from being influenced by prior knowledge about the subforum a post came from, a single post was exported with its text content and unique ID only, ensuring blindness to the post origin.

### 2.4 Annotation of forum posts

#### 2.4.1 Criteria

To create training material for the classifier, an annotation guide was developed for labelling forum posts as containing problem-gambling content (target posts), or containing gambling content only (non-target posts). Posts that could not be classified were labelled “inconclusive”. The guide consists of annotation instructions, as well as an annotation form in which the applicable criteria for a single forum post may be noted (see S1 File). The annotation guide is based on the criteria for gambling disorder as listed in the DSM-5 [1] and on the items from the Gambling Related Cognitions Scale (GRCS) [42]. The focus was put on coding the presence/absence of gambling-related problems and/or endorsement of gambling-related cognitions (i.e. target vs. non-target classification), rather than on a continuous measure of e.g. gambling severity. The temporal dimension of pathological gambling according to DSM-5 criteria (i.e., meeting a specific number of criteria within twelve months) was not taken into account in the annotation, as this cannot be reliably assessed on the basis of individual posts.

Problem gambling is often accompanied by gambling-related cognitive distortions, i.e. erroneous beliefs about gambling, such as the ability to predict or control gambling outcomes [4, 42]. To this end, the GRCS, a reliable and valid scale for the assessment of such beliefs, was additionally considered during annotation. The GRCS is a questionnaire developed to identify gambling-related cognitions in individuals that engage in gambling [42]. The instrument contains 23 items representing the 5 subscales gambling expectations, illusion of control, predictive control, inability to stop gambling, and interpretive bias.

For annotation purposes, the criteria of the DSM-5 and items of the GRCS were categorised into the three subdomains (1) pathological gambling, (2) gambling-related problems, and (3) gambling-related cognitive distortions. Items from both instruments covering similar characteristics were grouped together. Since the focus was on detecting problem-gambling content in individual posts, a post was considered a target, i.e. problem-gambling post if it contained at least one statement about the presence of pathological gambling behaviour, gambling-related problems, or gambling-related cognitive distortions. It was deemed irrelevant, whether the descriptions related to the author of a post or a related person (i.e. a friend or relative), or whether the author described current or past gambling-related problems. A post containing no such descriptions, but descriptions of gambling-related topics (e.g., casino complaints), was considered as non-target. Posts were also coded as targets if none of the items were explicitly reported in the descriptions, but the person clearly self-identified as being addicted to gambling, was actively seeking help or treatment for gambling disorder, or mentioned currently undergoing treatment.

#### 2.4.2 Annotations

**Fig 1:**
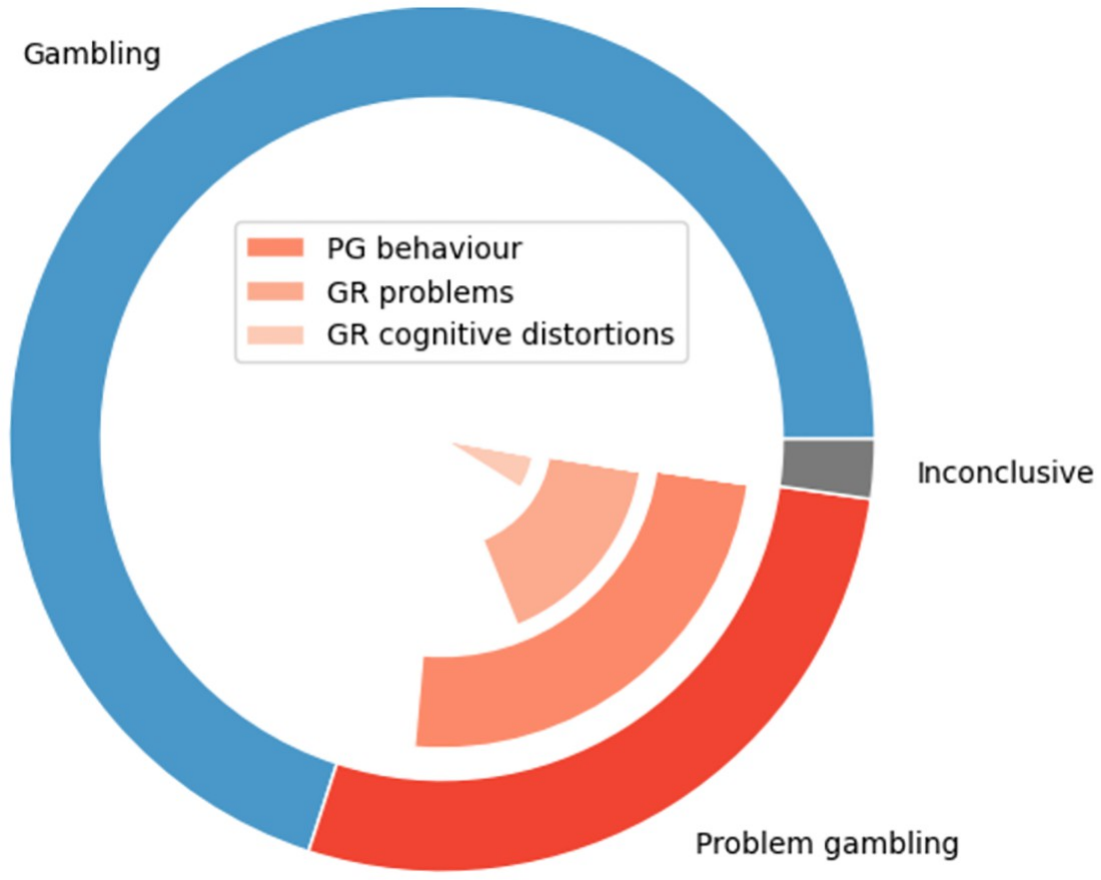
Categories assigned during annotation of the forum posts. In total, 504 posts were coded, yielding 348 posts labelled as gambling, 11 posts labelled as inconclusive (not included in the training set), and 138 posts labelled as problem gambling (PG). Seven posts were excluded for being unrelated to gambling (not depicted here). The problem gambling set contained 114 posts with signs of problem PG behaviour (e.g. increasing the stakes and chasing losses), 70 posts with signs of gambling-related (GR) problems (e.g. financial problems), and 23 posts with GR cognitive distortions (e.g. believing in lucky numbers, see also S1 File). Note that the three problem gambling subdomains are not mutually exclusive and therefore do not sum to the total number of PG posts.

In total, 504 posts were coded by manual and blind annotation using the annotation guide (see Fig 1). Seven posts were considered neither as target nor non-target posts and not included in the training set (one empty post, two posts from which the text content had been deleted by an administrator, and four posts that were not from regular users, but advertisements from universities to recruit study participants in the context of gambling addiction research). 11 posts were labelled as inconclusive and also not included in the training set, since it was not clear from the descriptions whether problem-gambling behaviour or cognitions were described. This was the case for posts in which persons asked questions about gambling addiction in a way that suggested they may be related to themselves, but without being specific about themselves, or posts in which the connection between described problems and gambling remained vague. 138 posts were annotated as targets, i.e., containing problem-gambling content, and 348 posts non-targets, i.e., containing other gambling content (see Table 2).

Among problem-gambling posts, frequently mentioned issues were financial difficulties as well as repeated unsuccessful attempts to stop gambling. Gambling-related cognitive distortions often manifested as the belief that specific numbers or changing numbers relates to the chances of winning (illusion of control), and the belief in hot streaks (predictive control). Gambling posts often contained questions about the trustworthiness of specific casinos or complaints about casinos (mostly problems with payouts after winnings). Examples of annotated target, non-target and inconclusive posts, respectively, are provided in S2 File.

### 2.5 Preprocessing and training data size

Manually screening the forum posts showed that capitalisation errors were frequent. Therefore, we lowercased all texts so as to prevent the model from treating such cases as different words. The texts were lowercased when tokenising using the BertTokenizer.

The forum posts also contained frequent punctuation errors. Removing punctuation is standard practice in NLP [43, 44]), and removing punctuation may reduce noise and thus improve the model’s ability to identify relationships between words. However, since many posts also contained emotional descriptions emphasised using punctuation (e.g. several consecutive exclamation marks), removing punctuation may result in the loss of important information related to emotional tone and urgency of describe issues with gambling. Since removing punctuation may have positive and negative effects, we decided to evaluate the impact of removing punctuation on the model’s performance. Hence, we compared classification performance for lowercased texts to lowercased texts from which punctuation characters were removed.

To consider the influence of the training data size, the model was fine-tuned using different set sizes. Specifically, the model was fine-tuned using one-half, two-thirds, and all target items, plus the same number of non-target items, respectively, yielding set sizes of 69/69, 92/92, and 138/138 (*N* non-target or gambling items, *N* target or problem-gambling items, see Table 2). When using subsamples of the annotated dataset (which contained more non-target than target items) the items were sampled with weighted subsampling based on text length to ensure that text length in tokens was not significantly different between target and non-target items (assessed with Welch’s t-tests for each dataset, with *p* < .05 for all tests). The model was also fine-tuned using all annotated items, which resulted in an imbalanced data set of size 348/138. Further, the model was fine-tuned using upsampling to match the number of problem-gambling items to the number of gambling items, yielding a balanced data set of size 348/348. For this purpose, the texts were converted to a matrix of term frequency-inverse document frequency (TF-IDF) features (a measure for the relevance of a word to a text document) in and then upsampled using the Synthetic Minority Over-Sampling Technique (SMOTE), as implemented in Imbalanced-Learn [45].

### 2.6 Model description

Bidirectional Encoder Representations from Transformers (BERT) is a pre-trained transformer-based ML model developed by the Google AI Language team in 2018 [46]. Pre-trained language models are trained on a huge corpus, thereby learning universal language representations, and then fine-tuned to a specific task in a subsequent step. For the present work, BERT was fine-tuned to the task of classifying forum posts as containing problem-gambling content vs. gambling content only. A large body of work has demonstrated that pre-trained language models outperform state-of-the-art models, such as recurrent neural networks, in a wide range of NLP tasks, and that these significantly reduce the amount of required training data (see, e.g., [46–49]). BERT enables solving NLP tasks in a supervised fashion when the dataset labelled for training is not large enough for achieving satisfactory classification performance using a model trained from scratch. Being a transformer model, BERT is based on the concept of self-attention. Attention describes a mechanism that calculates the importance of tokens relative to other tokens in a sequence (e.g., words in a sentence), or the likelihood that tokens appear together. For a detailed description of the model architecture, the reader is referred to [50].

### 2.7 Model implementation

BERT was implemented with Python (version 3.10.6) and the machine learning framework PyTorch (version 1.12.0) [51], and the Transformers library (version 4.21.0). For the current task, the German language model bert-base-german-uncased [52] was used. The German BERT model is based on BERT_BASE_ (12 encoder layers, 768 hidden units, 12-self-attention heads, 110 m parameters) and has been pre-trained on a German Wikipedia and OpenLegalData dump and news articles. For fine-tuning, a regression head (linear layer) was added on top of the output by implementing BertForSequenceClassification [53]. The maximum input sequence length was set to 512 tokens (maximum possible sequence length of the BERT model). Shorter texts were padded up to the maximum sequence length.

### 2.8 Fine-tuning and performance assessment

The choice of training hyperparameters was guided by the values reported as optimal for fine-tuning the original BERT model [46] and on the values reported in Bucur and colleagues for fine-tuning BERT to problem-gambling subreddits [31, 32], yielding a batch size of 16, using the Adam optimiser with weight decay (epsilon = 1e-08) for 2 epochs with a learning rate of 5e-5. Training was performed under Ubuntu (version 18.04.6 LTS) on a Dell Precision Workstation 5820 using a Nvidia Quadro RTX 5000 GPU. The model’s performance in classifying posts as either gambling or problem gambling was evaluated based on average accuracy, precision, recall, and F1 scores. While accuracy measures the fraction of correct predictions, it is not suitable for imbalanced data sets and does not allow for a comprehensive evaluation of the type of errors made. Precision may be considered a measure of quality, models with high precision may miss positive instances. Recall (also referred to as sensitivity) may be considered a measure of quantity, models with high recall tend to classify instances as belonging to the target class, thereby producing more false positives. Since, increasing precision usually comes at the cost of reduced recall, and vice versa, the F1 score takes into account both precision and recall. Performance was assessed for each fold using k-fold cross-validation with *k* = 5. This way, each of the data sets (see section 2.5) was randomly split into 5 subsets. In five turns, four of those were used for training, while the fifth was held out for testing.

### 2.9 Baseline model

To provide a baseline for the BERT classifier, we trained a support vector machine (SVM) based on a bag-of-words (BoW) representation to the task of classifying posts as either gambling or problem gambling posts. The approach was implemented with Python (version 3.10.6). First, we transformed the texts to a BoW representation (matrix of token counts) using the CountVectorizer class of the sklearn.feature_extraction.text module [54], building on a vocabulary considering the top 10% of features. The SVM classifier was implemented using the SVM class of the sklearn.svm module [54] using a linear kernel (λ = 1, *γ* = “scale”, with shrinking). As was the case for the main analysis, we trained the SVM classifier on different training set sizes (see section 2.5). All text was lowercased. Since the BoW model does not take into account punctuation, we did not consider the influence of removing punctuation. Classification performance was evaluated using k-fold cross-validation with *k* = 5, considering average accuracy, precision, recall and F1 score across all folds.

## 3 Results

### 3.1 Model validation

**Fig 2:**
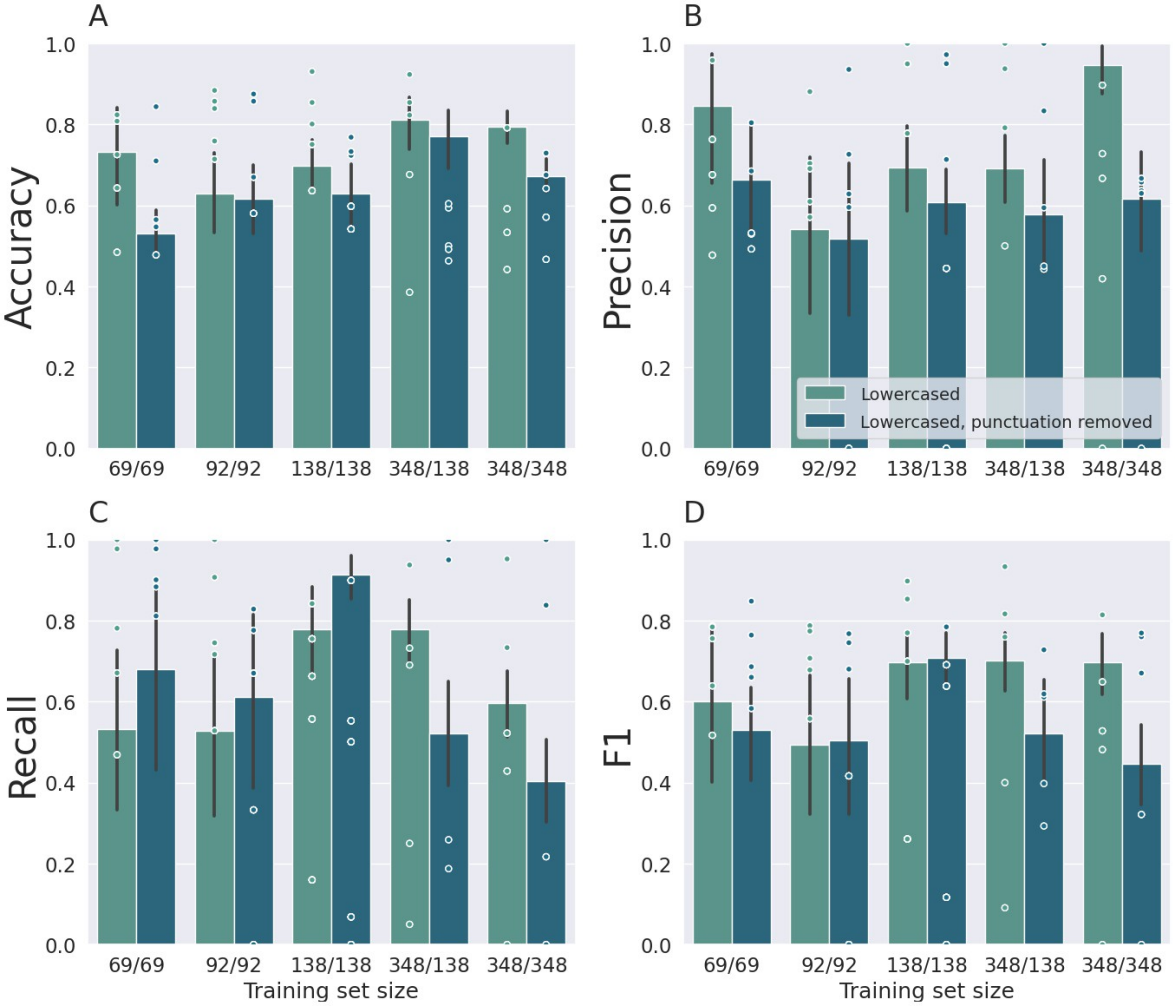
Accuracy (A), precision (B), recall (C) and F1 scores (D) of the BERT-based model, for each fold (points) and average accuracy with standard deviations across folds (bars), for each preprocessing pipeline and training set, sorted by the number of problem-gambling posts. The model was fine-tuned using one-half, two-thirds, and all target items, plus the same number of non-target items. The first number in the training set size denotes the count of non-target/gambling posts, the second number denotes the count of target/problem-gambling posts. The data with a training set size of 348/348 contain upsampled problem-gambling posts. For reasons of completeness, accuracy is depicted for all model runs, note, however, that accuracy is an inappropriate measure for unbalanced datasets (348/138). Following [55], precision, recall and F1 are zero, when a processed batch contains no target (i.e. problem-gambling post), but the classifier returns one or more targets.

Prediction performance of the model was compared based on the average performance scores across folds for each preprocessing pipeline and training set size (see Table 3 and Fig 2).

**Table 3:** Classification performance (means and standard deviations) for each preprocessing pipeline and training set size.

Model	G/PG items	Accuracy	Precision	Recall	F1
BERT with lowercasing	69/69	0.73 (0.19)	0.85 (0.29)	0.53 (0.33)	0.60 (0.31)
	92/92	0.63 (0.20)	0.54 (0.39)	0.52 (0.38)	0.49 (0.34)
	138/138	0.70 (0.15)	0.69 (0.25)	0.78 (0.25)	0.70 (0.19)
	348/138	0.81 (0.18)	0.69 (0.26)	0.78 (0.24)	0.70 (0.22)
	348/348	0.79 (0.13)	0.95 (0.21)	0.59 (0.21)	0.70 (0.26)
BERT with lowercasing and punctuation removal	69/69	0.53 (0.09)	0.66 (0.2)	0.68 (0.38)	0.53 (0.18)
	92/92	0.62 (0.17)	0.52 (0.37)	0.61 (0.43)	0.50 (0.33)
	138/138	0.63 (0.18)	0.61 (0.18)	0.91 (0.12)	**0.71** (0.15)
	348/138	0.77 (0.23)	0.58 (0.40)	0.52 (0.39)	0.52 (0.31)
	348/348	0.67 (0.13)	0.62 (0.43)	0.40 (0.35)	0.45 (0.34)
SVM with BoW	69/69	0.57 (0.06)	0.59 (0.09)	0.54 (0.13)	0.55 (0.07)
	92/92	0.53 (0.07)	0.53 (0.06)	0.50 (0.09)	0.51 (0.07)
	138/138	0.62 (0.06)	0.62 (0.04)	0.60 (0.10)	0.61 (0.07)
	348/138	0.72 (0.05)	0.53 (0.14)	0.36 (0.03)	0.43 (0.06)
	348/348	0.62 (0.12)	0.57 (0.09)	0.79 (0.26)	**0.66** (0.15)

*Note.* Detection rates are reported as means across folds. The data set with a size of 348/348 contains upsampled target/problem-gambling posts. For reasons of completeness, accuracy is listed for all model runs, note, however, that accuracy is an inappropriate measure for unbalanced datasets (348/138). The best score in terms of F1 score is shown in bold. G: gambling, PG: problem gambling, BERT: bidirectional encoder representations from transformers, SVM: support vector machine, BoW: bag-of-words.

#### 3.1.1 BERT model

First, we assessed for which training set size classification performance was above the baseline for binary classification, i.e. chance level (above 0.5% for balanced data sets). Performance was above the baseline for binary classification for all models with lowercasing only (keeping punctuation) and a training set size of 138/138 and larger. Accuracy, precision and F1 scores were higher for the models trained on larger training set sizes (see Fig 2A, 2B and 2D). The best model according to the F1 score proved to be the model using data with lowercasing and punctuation removal and a training set size of 138/138 (see Table 3, and Fig 2D). Accuracy and precision increased with training set size (see Fig 2A and 2B), while recall did not systematically vary with training set size. Precision and recall varied substantially between folds (see standard deviations in Fig 2B and 2C). The highest precision was found for the lowercased training data set with a training set size of 348/348 (see Fig 2B). Overall, keeping punctuation improved predictive performance. Upsampling problem-gambling posts (set size of 348/348) yielded an improvement in accuracy and precision, but not recall, compared to the balanced dataset (set size of 138/138) (see Fig 2A, 2B and 2D).

#### 3.1.2 Baseline model

The best classification performance with regard to F1 score (0.66) was obtained when training the SVM on the largest data set with 348 items per class (including upsampled problem gambling posts). Comparing the classification results of the SVM classifier to the BERT model showed that the BERT-based model performed better overall (see Table 3). For the smaller data set and the imbalanced data set, the SVM yielded comparatively poor classification performance. Precision was generally lower compared to the BERT model.

### 3.2 Error analysis

For the BERT-based model, we conducted an error analysis for the best model according to the F1 score (training set size of 138/138, lowercasing and punctuation removal, see Table 3). For this purpose, posts from all folds, for which the model’s predictions were false, were manually screened to identify potential systematic errors. For this model, recall was higher than precision (see Table 3 and Fig 2B and 2C). It was particularly noticeably that many of the false-positive predictions were made for posts including finance-related terms such as “money”, and “loss” (54% of false-positive predictions), and “bank”, “bank account”, “bank details”, “account”, “account statement”, and “account number” (29% of false-positive predictions). These posts were generally casino complaints (e.g. problems with payouts after winnings). This type of misclassification likely results from an overlap of content when writing casino complaints due to problems with payments, and content related to gambling-related financial problems, respectively. Further, many false-positive predictions were made for posts which included words such as “problem(s)” “help”, “support”, “warn” (91% of false-positive predictions). These words also frequently appeared in problem-gambling posts, since users often posted to seek for help and to warn other users from slipping into disordered gambling. False-negative predictions (i.e., problem-gambling posts misclassified as gambling posts) were in some cases even made for posts containing terms that are clearly associated with problem gambling, such as “gambling addiction” (36% of false-negative predictions) and “addiction” (43% of false-negative predictions). Thus, a post was not classified as problem gambling based solely on the presence of a term such as gambling addiction. This may arise from the fact that only a small proportion of the posts annotated as problem-gambling posts actually contained such terms. In most cases, problem-gambling behaviour or gambling-related cognitive distortions were described without explicitly naming the condition.

Some of the screened posts for which erroneous predictions were made contained terms related to actual or predicted gambling-related cognitive distortions. Automatically detecting problem gambling by means of gambling-related cognitive distortions is not trivial, since it is partly a matter of rather subtle distorted cognitive processes that have to become apparent from the posts. Automatic detection of gambling-related cognitive distortions represents a challenge for the model and it further appears that the occurrence of financial terms in the context of casino complaints may have impaired the model’s performance since financial problems are a central characteristic of problem gambling and a frequently discussed topic in the annotated posts.

## 4 Discussion

The aim of the current work was to identify potential signatures of problem gambling behaviour, gambling-related problems and cognitive distortions in online forum posts. Using data scraped from a major German online gambling discussion board, a subsample of posts was manually annotated in consideration of the criteria for gambling disorder as listed in the DSM-5 [1] and items of the GRCS [42]. The annotated data were then used to fine-tune a BERT model, a transformer-based pre-trained ML model for NLP [46], to the task of classifying posts into problem gambling (target category) vs. non-problem gambling content (non-target category).

### 4.1 Annotation of gambling forum posts

The annotation revealed that problem-gambling posts constituted the minority class. This reflects the observation that the number of posts in the gambling addiction subforum made up a small proportion of the overall forum. Problem-gambling posts mostly contained explicit descriptions of problems related to gambling and symptoms of disordered gambling. Frequently mentioned topics were financial difficulties and repeated unsuccessful attempts to stop gambling. However, problem-gambling posts were not exclusively found in the problem-gambling subforum. In posts from the other subforums, excluding the casino complaints and rules and guidelines subforum, frequently discussed topics were gambling experiences and strategies. Since gambling is, by definition, based on chance, writing about gambling strategies may reveal gambling-related cognitive distortions of the user. This was the case, for example, when users assumed that specific strategies, colours or numbers influenced the course of the game, or when users assumed that the game was based on skill, rather than chance, or followed a predictable pattern. Some characteristics of gambling disorder following the DSM-5 criteria such as the duration of symptoms [1] could of course not be reliably be determined from individual posts, and were therefore not considered. For future work, it might also be useful to code the severity of problem gambling content based on the number of described symptoms, and to annotate the posts using more granular categories, such as such as low-risk vs. high-risk problem gambling content for cases in which individuals describe following vs. not following self-imposed control strategies to regulate problem-gambling behaviour. Even if specific problem-gambling behaviour or symptoms are clearly reported, it must be kept in mind that this would obviously not suggest a clinical diagnosis. This requires a comprehensive structured clinical interview by a clinician. Furthermore, the level of analysis in the present approach is the individual post, and not the individual user. Needless to say, examining online posts cannot replace a comprehensive mental health assessment and diagnosis by an experienced practitioner [56, 57].

### 4.2 Model performance

Comparing the results from fine-tuning the BERT model to training a SVM based on a BoW representation on the same data demonstrated that the BERT model achieved markedly better classification performance. For the smaller data sets and the imbalanced data set, the SVM yielded comparatively poor classification performance. Notably, precision was generally lower compared to the BERT-based model. We consider the high precision a major strength of the BERT-based model, since high precision important with regard to the potential use case of screening large amounts of online texts, to prevent large amounts of false positives being generated.

SVMs generally work well for text categorisation [58] and may yield good performance for small data sets [59, 60]. Even though our training data sets were rather small, the performance for the two largest (balanced) data sets was above chance level for binary classification. SVMs are not feasible for large datasets, since training time easily becomes impractically long [61, 62]. Considering the potential application of our model, i.e. screening of large amounts of online texts for early detection of problem gambling signs, using a SVM classifier may be inefficient. Further, SVMs perform poorly in imbalanced datasets [63]. This was also the case for the present data. The worst classification performance with regard to precision, recall and F1 score was found when training the SVM on the imbalanced data set. In a realistic setting, there will class imbalance, since problem gambling content on the internet is comparatively rare compared to gambling or non-gambling related content. Therefore, the using a pre-trained model such as BERT, which requires comparatively little computation time and yielded higher precision in identifying problem gambling posts, proves to be more advantageous.

Looking at the effects of training set size on the BERT model showed that performance was satisfactory for lowercased training data with 138 or more items per class. When training the BERT model on additional, upsampled data, the model achieved high precision with a value of 0.95. Removing punctuation was not beneficial for model performance. On the contrary, it appears that punctuation conveyed some information relevant for distinguishing problem from non-problem gambling posts. This corresponds to the observation from the manual screenings that users often support descriptions of problems and negative emotions via punctuation [64, 65].

Bucur and colleagues [31, 32] took a similar approach to the one reported here, fine-tuning a pre-trained BERT model to detect signs of problem gambling. The authors scraped data from gambling addiction and problem-gambling subreddits, combining it with control data from other publicly available datasets. Comparing classification performance with regard to F1 score, the best model presented in the current work achieved a substantially better classification performance (yielding an F1 score of 0.71, compared to 0.27 and 0.41 [31, 32]).

Two key factors may explain the differences in classification performance. First, the training data from Bucur and colleagues [31, 32] were only loosely annotated (distant supervision), i.e. all posts from the chosen gambling addiction and problem-gambling subreddits were defined as positive class items, assuming that the majority of users posting in these subreddits exhibit problem gambling. The lack of manual annotation based on diagnostic criteria and gambling-related cognitive distortions in the construction of the training data can be seen critically in the context of construct validity and may have impaired prediction performance. Second, another key difference to the present work is the user-centred approach in collecting training data. From all unique users obtained from crawling the gambling-related subreddits, posts written in other, gambling-unrelated, subreddits were crawled to obtain both gambling-related and gambling-unrelated posts for the positive class. Negative class items were taken from control users from other openly available Reddit-based-datasets [31, 32]. By also including gambling-unrelated posts from the positive class users, the trained model may have missed important context or relevant information that could help distinguish signs of problematic gambling. Overall, the results of the current work demonstrate that by annotating forum posts based on diagnostic criteria and gambling-related cognitive distortions, satisfactory classification performance could be obtained with less training data compared to state-of-the-art work, using only 348 items per class as compared to processing a multiple of that (1,828 to 170,698 user writings, see [29–32]).

While there is room for improvement in terms of recall, considering a scenario where signs of problem gambling shall be detected from a plethora of discussion forum or social media posts requires high precision to avoid producing large amounts of false positives. For instance, if website operators are to initiate certain action cascades upon detecting possible signs of problem gambling using a ML application (e.g., contacting a user), this would only be feasible for precise predictions. Having to process large amounts of false positive cases would require many resources, and moreover, could lead to users feeling disturbed or monitored when being contacted frequently. Critically, while the approach may be suitable for detecting early signs of problem gambling, it would be highly problematic if someone were to draw far-reaching conclusions from individual posts. Therefore, ethical and privacy aspects must always be taken into consideration when implementing such models.

### 4.3 Model error patterns

To determine whether the errors that the model made in its predictions followed systematic patterns, an error analysis was conducted. Posts of the best model according to F1- score, for which false predictions were made during validation, were screened with regard to the (co)occurrence of specific terms. The error analysis essentially revealed that a substantial part of the posts for which false-positive predictions were made contained finance-related terms, and the word “problem(s)”. These types of gambling posts were mostly casino complaints featuring descriptions of problems with payouts at specific casinos. Likewise, problem-gambling posts frequently contained descriptions of (gambling-related) financial problems, which is why terms such as “problems” and “difficulties” were often used in combination with terms such as “bank” and “account balance”. The casino complaints subforum is the second largest subforum with regard to the absolute number of posts, and the overlap of terminology in problem-gambling posts and casino complaints posed a special challenge for the classifier. Considering the error patterns with regard to the transformer model architecture leads to revisiting the attention mechanism. The transformer model considers the relationships between tokens in an input sequence, including the context of the word “problem” when combined with finance-related words. The attention mechanism of the transformer model encodes a word based on other words in a sentence and indicates how strongly a word is associated with other words in the same sentence. Additionally, the positional encoding layer in both the encoder and decoders of the BERT model should account for the order of the tokens in the input sequence. This means that the model should be able to distinguish between different sequences of words, even if they contain the same words, based on their order in the input. However, it is possible that the model may still make errors in classifying text that contains the word “problem” in combination with finance-related words. One reason for this could be that the training data did not include enough examples of this specific pattern in casino complaints and problem-gambling posts, which may have led to the model not being able to learn the correct association between these words. Based on the described error patterns and the attention mechanism of the transformer model, it is possible that the model is over-emphasising the word “problem” and other finance-related terms in the input sequence while under-emphasising context (other tokens) that could help to differentiate between casino complaints and descriptions of gambling-related financial problems. To address this, it may be beneficial to add further training examples that reflect the nuances of the described problems more clearly.

Looking at false-negative predictions, it appeared that several posts were misclassified as gambling posts, even if they contained terms clearly associated with problem gambling, such as “gambling addiction” and “treatment”. These were mainly posts in which the authors clearly described that they were addicted to gambling, while not mentioning many symptoms or gambling-related cognitions. That the presence of problem gambling “keywords” is not sufficient for the model to predict problem gambling, may be related to the observation that in the majority of problem-gambling posts, problem-gambling behaviours or related cognitive distortions were described without explicitly mentioning the condition.

### 4.4 Limitations and ethical aspects

Overall, we used comparatively little data for classification, implying that a large part of the linguistic variance has not been explored. The implemented model is language-specific, and further, may not generalise to other platforms. Cross-validation was used to estimate how well the model would generalise. While overfitting may be detected using k-fold cross-validation, the performance estimates may still be overly optimistic if the model is overfitting. Different social media platforms may be associated with different posting behaviours and norms, which could affect the quality and representativeness of the data collected. The generalisability and applicability to texts from other online communities would have to be assessed in a next step. Further, the method for data collection may have introduced a sampling bias. Although survey studies indicate that individuals who demonstrate higher degrees of problem gambling tend to participate more actively in online gambling communities [15, 16], not all individuals who gamble participate in online gambling communities, and therefore, the training data may not be representative of the entire population of individuals who gamble.

A small fraction of posts was labelled as inconclusive and not included in the training set. In these cases, the descriptions provided were either to vague to classify the behaviour as either gambling or problem gambling, or users asked questions about problem gambling without being specific that the question related to themselves. The approach of not including inconclusive posts in the training data was taken to focus on clear and high-quality examples and to reduce noise since classification performance is strongly dependent on the quality and discriminatory power of the annotations and the amount of attribute noise [66]. However, excluding inconclusive posts may also have potential drawbacks. First, excluding posts limits the amount of available training data. Since only a small fraction of posts was labelled as inconclusive, this did not significantly impact on training set size. Second, the classifier may not perform as well in real-world scenarios containing inconclusive descriptions, which may mitigate its usefulness in practical applications. A potential outlook for further work could therefore be to include inconclusive posts as a third category for the machine learning classification. By creating a separate category for inconclusive posts, the classifier may be trained to distinguish between different types of posts, even if it cannot confidently assign them to the gambling or problem-gambling category. This may help to improve the robustness of the model for real-world scenarios. However, including a third category would also add complexity to the model and would require much more training data. Since inconclusive posts were comparatively rare, a substantially larger part of the data would have to be screened manually.

While modelling mental health status from social media usage may yield great benefits for monitoring site operators, some caution is also warranted when employing such techniques. Reviewing the literature, construct validity may frequently be questioned. It is not always clear, what the ground truth represents when annotation data with respect to signs of psychopathology [21]. As an example, Vaishnavi and colleagues [67] compared various ML-methods for the prediction of mental health, not indicating on what basis mental health was evaluated. In contrast, the annotation in the present study was performed manually based on clinically defined criteria, and we emphasise taking into account validated clinical criteria when implementing ML methods. Finally, considering such models for application in practice, it must be kept in mind that such methods bear the risk of algorithmic bias, i.e., systematic errors in machine-based decisions that create unfair outcomes for specific persons or groups [68, 69].

Using data obtained from public online forums offers the advantage of assembling large datasets without any interference from researchers, avoiding effects of direct observation. However, this method also requires significant ethical considerations. The role of “perceived privacy” within online communities should be considered [70]. For instance, if access to posts is restricted to registered users, it implies that contributors may perceive their communication as occurring in a private realm. If platform providers implement technical measures to limit data access, researchers should not circumvent these measures [40]. Adhering to these principles, we gathered publicly available data without bypassing any technical barriers.

### 4.5 Perspectives

Problem gambling is a public health concern and leads to serious psychological and economic consequences, such as depression and indebtedness for many of those affected [2]. According to a survey by the Federal Centre for Health Education in Germany (Bundeszentrale für gesundheitliche Aufklärung, BZgA) more than 400,000 individuals are estimated to exhibit problematic or pathological gambling behaviour in Germany in 2019 [71]. The increased availability of online gambling services and the legalisation of online gambling in more and more countries, including Germany, may pose a high risk for vulnerable individuals and may lead to changes in prevalence rates of problem gambling [72]. Since individuals frequently use social media to share information about health-related issues, thereby generating large amounts of data, the use of computational methods to automatically detect risk factors or changes in prevalence is becoming increasingly important. In Germany, the legalisation of online gambling as part of the GlüStV 2021 [10] may pose a special risk for vulnerable individuals. German operators of gambling halls are legally obliged to provide evidence of a social concept for gambling venues, which includes training on prevention measures and detection of addictive behaviours [10]. In contrast, online gambling is harder to monitor, and users can access illegal content more easily. As individuals with problematic gambling behaviour appear to be strongly involved in online communities [15, 16], the study of the content produced may help to indirectly measure changes in gambling behaviour. Automatically detecting signs of problem gambling may therefore be beneficial for monitoring activities of website operators in the context of player protection on the internet. Changes in gambling behaviour have been reported during the lockdown periods following the COVID-19 pandemic. A number of studies report an increase in online gambling, especially among vulnerable individuals [73, 74]. A potential application of the model employed here could be to determine changes in problem gambling during the COVID-19 lockdown phases in Germany. Automatic detection based on online communication could be particularly beneficial when on-site measurements of gambling behaviour are not possible.

### 4.6 Conclusion

We used ML to train a large language model to detect signs of problem gambling in online texts, finding higher prediction accuracy using manual annotation compared to previous work [29, 31, 32, 39]. While manual annotation is time-consuming, especially when the target items are rare, it seems to be an important requirement to produce clear, high-quality training data. In most cases, problem-gambling content was clearly distinguishable from other gambling content. Against the background of similar work [31, 32], satisfactory classification could be obtained with less training data compared to state-of-the-art work [29, 31, 32, 39]. Error analysis revealed that, due to an overlap in terminology (problem- and finance-related terms), distinguishing casino complaints from problem-gambling posts posed a challenge for the model. Still, the present work shows that automatically detecting signs of problem gambling from online texts using machine learning algorithms is feasible. The results confirm the viability of using BERT when the amount of available training data is limited [46] and demonstrate that satisfactory classification performance can be achieved by generating high-quality training material using manual annotation based on diagnostic criteria. Centrally, individual posts contain enough information to recognise signs of problem gambling, yielding potential applications of the model for preventive measures.

## Legal considerations

Web scraping for scientific purposes is legal in Germany if no access restrictions are circumvented [37, 75]. The data scraped from the website were generally accessible at the time of data collection and we did not disregard any technical measures designed to prevent web scraping. Data collection did not cause technical damage to the website operator. Our research serves non-commercial purposes only. We do not hold the right to share the data and adhere to user confidentiality and compliance with German laws.

## Supporting information

S1 File Annotation guide and form. (PDF)

S2 File Annotation examples. (PDF)

## References

[1] American Psychiatric Association. Diagnostisches und statistisches Manual psychischer Störungen–DSM-5 (R). Hogrefe Verlag; 2014.

[2] Fong TW. The biopsychosocial consequences of pathological gambling. Psychiatry (Edgmont). 2005;2(3):22. 21179626 PMC3004711

[3] Goodie AS, Fortune EE. Measuring cognitive distortions in pathological gambling: review and meta-analyses. Psychology of Addictive Behaviors. 2013;27(3):730. doi: 10.1037/a0031892 23438249

[4] Johansson A, Grant JE, Kim SW, Odlaug BL, Götestam KG. Risk factors for problematic gambling: A critical literature review. Journal of Gambling Studies. 2009;25(1):67–92. doi: 10.1007/s10899-008-9088-6 18392670

[5] World Health Organization. ICD-11: International classification of diseases 11th revision. Retrieved September. 2018;6:2021.

[6] Griffiths M. Problem gambling in Europe: what do we know? Casino & Gaming International. 2010;6(2):81–84.

[7] Sundqvist K, Rosendahl I. Problem gambling and psychiatric comorbidity—risk and temporal Sequencing among women and men: Results from the Swelogs case–control study. Journal of Gambling Studies. 2019;35(3):757–771. doi: 10.1007/s10899-019-09851-2 31025162 PMC6679831

[8] Lancet T. Problem gambling is a public health concern; 2017. 10.1016/S0140-6736(17)32333-4 28872015

[9] Staatsvertrag zum Glücksspielwesen in Deutschland (Glücksspielstaatsvertrag—GlüStV);. Available from: https://gluecksspiel.uni-hohenheim.de/fileadmin/einrichtungen/gluecksspiel/Staatsvertrag/GlueStV.pdf.

[10] Staatsvertrag zur Neuregulierung des Glücksspielwesens in Deutschland (Glücksspielstaatsvertrag 2021—GlüStV 2021);. Available from: https://gesetze.berlin.de/bsbe/document/aiz-jlr-Gl%C3%BCStVtrBE2021rahmen%4020210701.

[11] Krumpal I. Determinants of social desirability bias in sensitive surveys: a literature review. Quality & Quantity. 2013;47(4):2025–2047. doi: 10.1007/s11135-011-9640-9

[12] Bradburn NM, Rips LJ, Shevell SK. Answering autobiographical questions: The impact of memory and inference on surveys. Science. 1987;236(4798):157–161. doi: 10.1126/science.3563494 3563494

[13] Griffiths MD. The use of online methodologies in data collection for gambling and gaming addictions. International journal of mental health and addiction. 2010;8(1):8–20. doi: 10.1007/s11469-009-9209-1

[14] Sirola A, Kaakinen M, Savolainen I, Paek HJ, Zych I, Oksanen A. Online identities and social influence in social media gambling exposure: A four-country study on young people. Telematics and Informatics. 2021;60:101582. doi: 10.1016/j.tele.2021.101582

[15] Sirola A, Kaakinen M, Oksanen A. Excessive gambling and online gambling communities. Journal of Gambling Studies. 2018;34(4):1313–1325. doi: 10.1007/s10899-018-9772-0 29623505

[16] Sirola A, Kaakinen M, Savolainen I, Oksanen A. Loneliness and online gambling-community participation of young social media users. Computers in Human Behavior. 2019;95:136–145. doi: 10.1016/j.chb.2019.01.023

[17] Lesieur HR, Blume SB. The South Oaks Gambling Screen (SOGS): a new instrument for the identification of pathological gamblers. Am J Psychiatry. 1987;144(9):1184–8. doi: 10.1176/ajp.144.9.1184 3631315

[18] Caputo A. Sharing problem gamblers’ experiences: A text analysis of gambling stories via online forum. Mediterranean Journal of Clinical Psychology. 2015;3(1).

[19] Im EO, Chee W. An online forum as a qualitative research method: practical issues. Nursing Research. 2006;55(4):267. doi: 10.1097/00006199-200607000-00007 16849979 PMC2491331

[20] Chancellor S, De Choudhury M. Methods in predictive techniques for mental health status on social media: a critical review. NPJ Digital Medicine. 2020;3(1):1–11. doi: 10.1038/s41746-020-0233-7 32219184 PMC7093465

[21] Merchant RM, Asch DA, Crutchley P, Ungar LH, Guntuku SC, Eichstaedt JC, et al. Evaluating the predictability of medical conditions from social media posts. PLOS ONE. 2019;14(6):e0215476. doi: 10.1371/journal.pone.0215476 31206534 PMC6576767

[22] Garner H. Engineering in genomics: the emerging in-silico scientist; how text-based bioinformatics is bridging biology and artificial intelligence. IEEE Engineering in Medicine and Biology Magazine. 2004;23(2):87–93. doi: 10.1109/MEMB.2004.1310989 15264477

[23] Névéol A, Dalianis H, Velupillai S, Savova G, Zweigenbaum P. Clinical natural language processing in languages other than english: opportunities and challenges. Journal of Biomedical Semantics. 2018;9(1):1–13. doi: 10.1186/s13326-018-0179-8 29602312 PMC5877394

[24] Pennebaker JW, King LA. Linguistic styles: language use as an individual difference. Journal of Personality and Social Psychology. 1999;77(6):1296. doi: 10.1037/0022-3514.77.6.1296 10626371

[25] Parapar J, Martín-Rodilla P, Losada DE, Crestani F. Overview of eRisk at CLEF 2021: Early Risk Prediction on the Internet (Extended Overview). CLEF (Working Notes). 2021; p. 864–887.

[26] Parapar J, Martín-Rodilla P, Losada DE, Crestani F. Overview of erisk 2022: Early risk prediction on the internet. In: Experimental IR Meets Multilinguality, Multimodality, and Interaction: 13th International Conference of the CLEF Association, CLEF 2022, Bologna, Italy, September 5–8, 2022, Proceedings. Springer; 2022. p. 233–256.

[27] Yates A, Cohan A, Goharian N. Depression and self-harm risk assessment in online forums. arXiv preprint arXiv:170901848. 2017;.

[28] Losada DE, Crestani F. A test collection for research on depression and language use. In: Experimental IR Meets Multilinguality, Multimodality, and Interaction: 7th International Conference of the CLEF Association, CLEF 2016, Évora, Portugal, September 5-8, 2016, Proceedings 7. Springer; 2016. p. 28–39.

[29] Loyola JM, Burdisso S, Thompson H, Cagnina LC, Errecalde M. UNSL at eRisk 2021: A Comparison of Three Early Alert Policies for Early Risk Detection. In: CLEF (working notes); 2021. p. 992–1021.

[30] Fabregat H, Duque A, Araujo L, Martinez-Romo J. UNED-NLP at eRisk 2022: Analyzing gambling disorders in social media using approximate nearest neighbors. Proceedings of the Working Notes of CLEF. 2022;.

[31] Bucur AM, Cosma A, Dinu LP. Early risk detection of pathological gambling, self-harm and depression using BERT. arXiv preprint arXiv:210616175. 2021;.

[32] Bucur AM, Cosma A, Dinu LP, Rosso P. An end-to-end set transformer for user-level classification of depression and gambling disorder. arXiv preprint arXiv:220700753. 2022;.

[33] Chan B, Möller T, Pietsch M, Soni T, Yeung CM. German BERT. URL: https://deepset.ai/german-bert. 2019;.

[34] Van Rossum G, Drake FL. Python 3 Reference Manual. Scotts Valley, CA: CreateSpace; 2009.

[35] Richardson L. Beautiful soup documentation. April. 2007;.

[36] Hipp RD. SQLite; 2020. Available from: https://www.sqlite.org/index.html.

[37] für Sozial R, et al. Big Data in den Sozial-, Verhaltens-und Wirtschaftswissenschaften: Datenzugang und Forschungsdatenmanagement. Mit Gutachten “Web Scraping in der unabhängigen wissenschaftlichen Forschung”. RatSWD Output; 2019.

[38] Fino E, Hanna-Khalil B, Griffiths MD. Exploring the public’s perception of gambling addiction on Twitter during the COVID-19 pandemic: Topic modelling and sentiment analysis. Journal of addictive diseases. 2021;39(4):489–503. doi: 10.1080/10550887.2021.1897064 33781174

[39] Maupomé D, Armstrong MD, Rancourt F, Soulas T, Meurs MJ. Early Detection of Signs of Pathological Gambling, Self-Harm and Depression through Topic Extraction and Neural Networks. In: CLEF (working notes); 2021. p. 1031–1045.

[40] Landers RN, Brusso RC, Cavanaugh KJ, Collmus AB. A primer on theory-driven web scraping: Automatic extraction of big data from the Internet for use in psychological research. Psychological methods. 2016;21(4):475. doi: 10.1037/met0000081 27213980

[41] Bird S, Klein E, Loper E. Natural language processing with Python: analyzing text with the natural language toolkit. O’Reilly Media, Inc.; 2009.

[42] Raylu N, Oei TP. The Gambling Related Cognitions Scale (GRCS): Development, confirmatory factor validation and psychometric properties. Addiction. 2004;99(6):757–769. doi: 10.1111/j.1360-0443.2004.00753.x 15139874

[43] Nesca M, Katz A, Leung CK, Lix LM. A scoping review of preprocessing methods for unstructured text data to assess data quality. International Journal of Population Data Science. 2022;7(1). doi: 10.23889/ijpds.v6i1.1757 37670734 PMC10476151

[44] Palomino MA, Aider F. Evaluating the effectiveness of text pre-processing in sentiment analysis. Applied Sciences. 2022;12(17):8765. doi: 10.3390/app12178765

[45] Lemaître G, Nogueira F, Aridas CK. Imbalanced-learn: A Python Toolbox to Tackle the Curse of Imbalanced Datasets in Machine Learning. Journal of Machine Learning Research. 2017;18(17):1–5.

[46] Devlin J, Chang MW, Lee K, Toutanova K. BERT: Pre-training of deep bidirectional transformers for language understanding. arXiv preprint arXiv:181004805. 2018;.

[47] Dai Z, Yang Z, Yang Y, Carbonell J, Le QV, Salakhutdinov R. Transformer-xl: Attentive language models beyond a fixed-length context. arXiv preprint arXiv:190102860. 2019;.

[48] Lakew SM, Cettolo M, Federico M. A comparison of transformer and recurrent neural networks on multilingual neural machine translation. arXiv preprint arXiv:180606957. 2018;.

[49] Wolf T, Debut L, Sanh V, Chaumond J, Delangue C, Moi A, et al. Transformers: State-of-the-art natural language processing. In: Proceedings of the 2020 conference on empirical methods in natural language processing: system demonstrations; 2020. p. 38–45.

[50] Vaswani A, Shazeer N, Parmar N, Uszkoreit J, Jones L, Gomez AN, et al. Attention is all you need. Advances in Neural Information Processing Systems. 2017;30.

[51] Paszke A, Gross S, Massa F, Lerer A, Bradbury J, Chanan G, et al. PyTorch: An Imperative Style, High-Performance Deep Learning Library. In: Advances in Neural Information Processing Systems 32. Curran Associates, Inc.; 2019. p. 8024–8035. Available from: http://papers.neurips.cc/paper/9015-pytorch-an-imperative-style-high-performance-deep-learning-library.pdf.

[52] dbmdz/bert-base-german-uncased · Hugging Face—huggingface.co;. https://huggingface.co/dbmdz/bert-base-german-uncased.

[53] Hugging Face. BERT For Sequence Classification;. Available from: https://huggingface.co/docs/transformers/v4.26.0/en/model_doc/bert#transformers.BertForSequenceClassification.

[54] Pedregosa F, Varoquaux G, Gramfort A, Michel V, Thirion B, Grisel O, et al. Scikit-learn: Machine learning in Python. the Journal of machine Learning research. 2011;12:2825–2830.

[55] Röder M, Usbeck R, Ngonga Ngomo AC. Gerbil–benchmarking named entity recognition and linking consistently. Semantic Web. 2018;9(5):605–625. doi: 10.3233/SW-170286

[56] Karches KE. Against the iDoctor: why artificial intelligence should not replace physician judgment. Theoretical Medicine and Bioethics. 2018;39(2):91–110. doi: 10.1007/s11017-018-9442-3 29992371

[57] Hallowell N, Badger S, McKay F, Kerasidou A, Nellåker C. Democratising or disrupting diagnosis? Ethical issues raised by the use of AI tools for rare disease diagnosis. SSM-Qualitative Research in Health. 2023;3:100240. doi: 10.1016/j.ssmqr.2023.100240 37426704 PMC10323712

[58] Joachims T. Text categorization with support vector machines: Learning with many relevant features. In: European conference on machine learning. Springer; 1998. p. 137–142.

[59] Han Y, Cui P, Zhang Y, Zhou R, Yang S, Wang J. Remote sensing sea ice image classification based on multilevel feature fusion and residual network. Mathematical Problems in Engineering. 2021;2021:1–10. doi: 10.1155/2021/5585398

[60] Althnian A, AlSaeed D, Al-Baity H, Samha A, Dris AB, Alzakari N, et al. Impact of dataset size on classification performance: an empirical evaluation in the medical domain. Applied Sciences. 2021;11(2):796. doi: 10.3390/app11020796

[61] Birzhandi P, Kim KT, Youn HY. Reduction of training data for support vector machine: a survey. Soft Computing. 2022;26(8):3729–3742. doi: 10.1007/s00500-022-06787-5

[62] Cervantes J, Li X, Yu W, Li K. Support vector machine classification for large data sets via minimum enclosing ball clustering. Neurocomputing. 2008;71(4-6):611–619. doi: 10.1016/j.neucom.2007.07.028

[63] Batuwita R, Palade V. Class imbalance learning methods for support vector machines. Imbalanced learning: Foundations, algorithms, and applications. 2013; p. 83–99. doi: 10.1002/9781118646106.ch5

[64] Hakami SAA, Hendley RJ, Smith P. Emoji Sentiment Roles for Sentiment Analysis: A Case Study in Arabic Texts. In: Proceedings of the The Seventh Arabic Natural Language Processing Workshop (WANLP); 2022. p. 346–355.

[65] Shoeb AAM, Raji S, de Melo G. EmoTag–Towards an emotion-based analysis of emojis. In: Proceedings of the International Conference on Recent Advances in Natural Language Processing (RANLP 2019); 2019. p. 1094–1103.

[66] Zhu X, Wu X. Class noise vs. attribute noise: A quantitative study. Artificial intelligence review. 2004;22:177–210. doi: 10.1007/s10462-004-0751-8

[67] Vaishnavi K, Kamath UN, Rao BA, Reddy NS. Predicting mental health illness using machine learning algorithms. In: Journal of Physics: Conference Series. vol. 2161. IOP Publishing; 2022. p. 012021.

[68] Delgado J, de Manuel A, Parra I, Moyano C, Rueda J, Guersenzvaig A, et al. Bias in algorithms of AI systems developed for COVID-19: A scoping review. Journal of Bioethical Inquiry. 2022;19(3):407–419. doi: 10.1007/s11673-022-10200-z 35857214 PMC9463236

[69] Walsh CG, Chaudhry B, Dua P, Goodman KW, Kaplan B, Kavuluru R, et al. Stigma, biomarkers, and algorithmic bias: recommendations for precision behavioral health with artificial intelligence. JAMIA open. 2020;3(1):9–15. doi: 10.1093/jamiaopen/ooz054 32607482 PMC7309258

[70] Eysenbach G, Till JE. Ethical issues in qualitative research on internet communities. Bmj. 2001;323(7321):1103–1105. doi: 10.1136/bmj.323.7321.1103 11701577 PMC59687

[71] Banz M. Glücksspielverhalten und Glücksspielsucht in Deutschland. Ergebnisse des Surveys 2019 und Trends. BzgA-Forschungsbericht; 2019.

[72] Gainsbury SM. Online gambling addiction: the relationship between internet gambling and disordered gambling. Current Addiction Reports. 2015;2(2):185–193. doi: 10.1007/s40429-015-0057-8 26500834 PMC4610999

[73] Price A. Online gambling in the midst of COVID-19: a nexus of mental health concerns, substance use and financial stress. International Journal of Mental Health and Addiction. 2020; p. 1–18. doi: 10.1007/s11469-020-00366-1 32837444 PMC7357671

[74] Sallie SN, Ritou VJ, Bowden-Jones H, Voon V. Assessing online gaming and pornography consumption patterns during COVID-19 isolation using an online survey: Highlighting distinct avenues of problematic internet behavior. Addictive Behaviors. 2021;123:107044. doi: 10.1016/j.addbeh.2021.107044 34311186 PMC8434429

[75] Klawonn T. Urheberrechtliche Grenzen des Web Scrapings (Web Scraping under German Copyright Law). Available at SSRN 3491192. 2019;.

