# Supplementary material for: Automatic detection of problem-gambling signs from online texts using large language models
Source: PLOS Digit Health. 2024 Sep 25;3(9):e0000605. doi: 10.1371/journal.pdig.0000605 (PMC11423982; doi:10.1371/journal.pdig.0000605)
Supplement: S1 File — (PDF) [file pdig.0000605.s001.pdf]

# ANNOTATIONSLEITFADEN

Anhand dieses Annotationsleitfadens und des dazugehörigen Annotationsbogens sollen Schilderungen zu pathologischem Glücksspielen, glücksspielbezogenen Problemen sowie glücksspielbezogenen kognitiven Verzerrungen in Forenbeiträgen kodiert werden. Der Annotationsleitfaden basiert auf der 5. Auflage der Kriterien des *Diagnostic and Statistical Manual of Mental Disorders* (DSM) zu pathologischem Glücksspielen (APA, 2014), sowie der auf der *Gambling Related Cognitions Scale* (GRCS,  Raylu & Oei, 2004).

Laut DSM-5 liegt pathologisches Glücksspielen vor, wenn mindestens vier der folgenden Kriterien innerhalb eines Zeitraums von zwölf Monaten vorliegen:

1. Notwendigkeit des Glücksspielens mit immer höheren Einsätzen, um eine gewünschte Erregung zu erreichen.
2. Unruhe und Reizbarkeit beim Versuch, das Glücksspielen einzuschränken oder aufzugeben.
3. Wiederholte erfolglose Versuche, das Glückspielen zu kontrollieren, einzuschränken oder aufzugeben.
4. Starke gedankliche Eingenommenheit durch Glücksspielen (zum Beispiel starke Beschäftigung mit dem gedanklichem Nacherleben vergangener Spielerfahrungen, mit dem Verhindern oder Planen der nächsten Spielunternehmung oder mit dem Nachdenken über Wege, Geld zum Glücksspielen zu beschaffen).
5. Häufiges Glücksspielen in belastenden Gefühlszuständen (zum Beispiel bei Hilflosigkeit, Schuldgefühlen, Angst oder depressiver Stimmung).
6. Rückkehr zum Glücksspielen am nächsten Tag, um Verluste auszugleichen (dem Verlust „hinterherjagen“ [„Chasing“]).
7. Belügen anderer, um das Ausmaß der Verstrickung in das Glücksspielen zu vertuschen.
8. Gefährdung oder Verlust einer wichtigen Beziehung, eines Arbeitsplatzes, von Ausbildungs- oder Aufstiegschancen aufgrund des Glücksspielens.
9. Sich auf die finanzielle Unterstützung von anderen verlassen, um die durch das Glücksspielen verursachte finanzielle Notlage zu überwinden.

Das DSM-5 kodiert auch den Schweregrad der Glücksspielstörung (leicht bis schwer). Bei der Annotation bleibt dies unberücksichtigt, da die Forumsnutzer:innen möglicherweise nur manche ihrer Probleme in einem Beitrag beschreiben und die Beschreibungen daher unvollständig sein können (beispielsweise kann sich ein/e Nutzer:in darauf konzentrieren, Rat zu schweren finanziellen Problemen einzuholen, ohne andere Probleme zu erwähnen). Auch die zeitliche Dimension pathologischen Glücksspielens der Kriterien des DSM-5 bleibt bei der Annotation unberücksichtigt, da diese in den meisten Fällen nicht zuverlässig auf Grundlage von Schilderungen aus Forenbeiträgen beurteilt werden kann, und vielmehr das Vorliegen von glücksspielbezogenen Problemen und Kognitionen im Vordergrund steht, nicht jedoch das Erfüllen aller diagnostischen Kriterien.

Problematisches Glücksspiel geht häufig mit glücksspielbezogenen kognitiven Verzerrungen einher, d. h. falschen Überzeugungen über das Glücksspiel, wie beispielsweise die Annahme, über die Fähigkeit zu verfügen, Spielergebnisse vorherzusagen oder zu kontrollieren (Johansson et al., 2009, Raylu & Oei, 2004). Da die DSM-5-Kriterien hauptsächlich glücksspielbezogenes Verhalten sowie Probleme abdecken, wurden

außerdem Items der GRCS bei der Entwicklung des Annotationleitfadens berücksichtigt, da dieses Instrument stärker kognitive Aspekte problematischen Glücksspielens abdeckt. Die GRCS enthält 23 Items zu den 5 Subskalen *gambling expectancies* (dt: „Erwartungen an das Glücksspiel“), *illusion of control* (dt: Kontrollillusion), *predictive control* (dt: prädiktive Kontrolle), *inability to stop gambling* (dt: Unfähigkeit, mit dem Glücksspiel aufzuhören), *interpretive bias* (dt: Interpretationsverzerrung). Die Items werden auf einer 7-stufigen Likert-Scala von 1 = *strongly disagree* (dt: stimme überhaupt nicht zu) bis 7 = *strongly agree* (dt: stimme voll zu) beantwortet. Die GRCS weist gute psychometrische Eigenschaften auf (Del Prete et al., 2017; Donati et al., 2015; Smith et al., 2016) und findet häufig Anwendung zur Diagnostik und Evaluation von Behandlungen, sowie in den klinischen Neurowissenschaften (Kayser et al., 2017; Smith et al., 2016; Worhunsky et al., 2014).

Zur Annotation wurden Items der Kriterien des DSM-5 sowie des GRCS den drei Teilbereichen Verhalten, Probleme und Kognitionen zugeordnet und zusammengefasst: (1) pathologisches Glücksspielen, (2) glücksspielbezogene Probleme, (3) und glücksspielbezogene kognitive Verzerrungen. Items aus beiden Instrumenten, die ähnliche Merkmale abdecken, wurden zusammengefasst. Beispielsweise finden die Items "Es ist schwierig, mit dem Glücksspiel aufzuhören, da ich keine Kontrolle habe." (GRCS) und "Wiederholte erfolglose Versuche, das Glückspielen zu kontrollieren, einzuschränken oder aufzugeben." (DSM-5) sich in der Kategorie *pathologisches Glücksspiel* des Annotationsleitfadens und werden als gemeinsames Item beschrieben. Merkmale, die nicht von beiden Instrumenten erfasst werden, sind einzeln aufgeführt (beispielsweise wurden die GRCS-Items zu interpretativen Verzerrungen in zusammengefasster Form unter *glücksspielbezogene kognitiven Verzerrungen* aufgeführt). Items zur Subskala *inability to stop gambling* aus dem GRCS (Raylu & Oei, 2004) wurden unter „glücksspielbezogene kognitive Verzerrungen“ nicht aufgeführt, da diese Eigenschaften bereits durch „pathologisches Glücksspielen“ (angelehnt an die Kriterien des DSM-5) abgedeckt werden.

## Hinweise zur Kodierung

Im Annotationsbogen sollen Items kodiert werden, die eindeutig aus den Schilderungen in einem Forenbeitrag hervorgehen. Beispiele hierfür sind:

Finanzielle Schwierigkeiten (glücksspielbezogene Probleme):

- „Ich spielte immer weiter und von meinen 1400 € Netto im Monat blieb kaum was übrigen [sic!]. Ich verspielte immer wieder hunderte von Euros.“

Verlassen auf finanzielle Unterstützung durch andere, um die durch das Glücksspielen verursachte finanzielle Notlage zu überwinden (glücksspielbezogene Probleme):

- „Die Rechnungen musste dann mein Vater für mich bezahlen. Inzwischen habe ich ca. 3000 € Schulden bei ihm.“

Wiederholte erfolglose Versuche, das Glücksspielen zu kontrollieren, einzuschränken oder aufzugeben (pathologisches Glücksspielen):

- „Man verliert die Kontrolle.“

Es gibt eine gewisse Kontrolle über die Vorhersage von Gewinnen (glücksspielbezogene kognitive Verzerrungen, genauer prädiktive Kontrolle):

- „ … dass Casino Software absolut steuerbar ist.“

Es gibt Zeiten, in denen man Glück hat und deshalb wird zu diesen Zeiten gespielt (glücksspielbezogene kognitive Verzerrungen, genauer prädiktive Kontrolle):

- „Sprich, ich habe 4 Tage en suite um die gleiche Zeit, mit dem gleichen Einsatz das gleiche Spiel gespielt und 4 mal zur selben Minute die Freespins erhalten.“

Beiträge sollen außerdem kodiert werden, wenn keines der Items explizit beschrieben wurde, die schreibende Person sich jedoch selbst als spielsüchtig bezeichnet, Hilfe für Spielsucht sucht, oder sich laut Schilderung wegen problematischem Glücksspiel in Behandlung befindet.

Enthält ein Forenbeitrag ausschließlich Schilderungen zum Glücksspiel sowie andere Inhalte, wird nicht kodiert. Beispiele hierfür sind:

- „Ich möchte eindringlich vor diesem Casino warnen!!“

- „Hier meine Meinung zum Platincasino bei dem ich seit drei Tagen angemeldet bin.“).

## Referenzen


American Psychiatric Association. (2014). *Diagnostisches und statistisches manual psychischer Störungen–DSM-5®*. Hogrefe Verlag.

Del Prete, F., Steward, T., Navas, J. F., Fernandez-Aranda, F., Jimenez-Murcia, S., Oei, T. P., & Perales, J. C. (2017). The role of affect-driven impulsivity in gambling cognitions: A convenience-sample study with a Spanish version of the Gambling-Related Cognitions Scale. *Journal of Behavioral Addictions*, *6*(1), 51-63.

Donati, M. A., Ancona, F., Chiesi, F., & Primi, C. (2015). Psychometric properties of the Gambling Related Cognitions Scale (GRCS) in young Italian gamblers. *Addictive Behaviors*, *45*, 1-7.

Johansson A, Grant JE, Kim SW, Odlaug BL, Götestam KG. Risk factors for problematic gambling: A critical literature review. *Journal of Gambling Studies*. 2009;25(1):67-92.

Kayser, A. S., Vega, T., Weinstein, D., Peters, J., & Mitchell, J. M. (2017). Right inferior frontal cortex activity correlates with tolcapone responsivity in problem and pathological gamblers. *NeuroImage: Clinical*, *13*, 339-348.

Raylu, N., & Oei, T. P. (2004). The Gambling Related Cognitions Scale (GRCS): Development, confirmatory factor validation and psychometric properties. *Addiction*, *99*(6), 757-769.

Smith, D., Woodman, R., Drummond, A., & Battersby, M. (2016). Exploring the measurement structure of the Gambling Related Cognitions Scale (GRCS) in treatment-seekers: A Bayesian structural equation modelling approach. *Psychiatry research*, *237*, 90-96.

Worhunsky, P. D., Malison, R. T., Rogers, R. D., & Potenza, M. N. (2014). Altered neural correlates of reward and loss processing during simulated slot-machine fMRI in pathological gambling and cocaine dependence. *Drug and alcohol dependence*, *145*, 77-86.

# ANNOTATIONSBOGEN

ID [ ]

| | Vorhanden |
|---|---|
| **Pathologisches Glücksspielen** | |
| Notwendigkeit des Glücksspielens mit immer höheren Einsätzen | ☐ |
| Unruhe und Reizbarkeit bei dem Versuch, das Glücksspielen einzuschränken oder aufzugeben | ☐ |
| Wiederholte erfolglose Versuche, das Glücksspielen zu kontrollieren, einzuschränken oder aufzugeben | ☐ |
| Starke gedankliche Eingenommenheit durch Glücksspielen | ☐ |
| Häufiges Glücksspielen in belastenden Gefühlszuständen | ☐ |
| Rückkehr zum Glücksspielen um Verluste auszugleichen | ☐ |
| Belügen anderer, um das Ausmaß der Verstrickung in das Glücksspielen zu vertuschen | ☐ |

| | |
|---|---|
| **Glücksspielbezogene Probleme** | |
| Gefährdung oder Verlust einer wichtigen Beziehung, eines Arbeitsplatzes, von Ausbildungs- oder Aufstiegschancen aufgrund des Glücksspielens | ☐ |
| Finanzielle Schwierigkeiten | ☐ |
| Verlassen auf finanzielle Unterstützung durch andere, um die durch das Glücksspielen verursachte finanzielle Notlage zu überwinden | ☐ |

| | |
|---|---|
| **Glücksspielbezogene kognitive Verzerrungen** | |
| Erwartungen an das Glücksspiel | |
| Glückspielen macht glücklich und/oder macht die Zukunft besser und/oder reduziert Anspannung und Stress | ☐ |
| Kontrollillusionen | |
| Spezifische Farben und Zahlen, Objekte, sowie Rituale und Verhaltensweisen (bspw. auch Beten) erhöhen die Gewinnchancen | ☐ |
| Prädiktive Kontrolle | |
| Auf Verluste beim Glücksspiel folgt zwangsläufig eine Reihe von Gewinnen, und/oder wenn einmal gewonnen wurde, wird später bestimmt wieder gewonnen | ☐ |
| Aus einer Reihe von Niederlagen kann gelernt werden, was wiederum hilft, später zu gewinnen | ☐ |
| Es gibt Zeiten, in denen man Glück hat und deshalb wird zu diesen Zeiten gespielt | ☐ |
| Es gibt eine gewisse Kontrolle über die Vorhersage von Gewinnen | ☐ |

| | |
|---|---|
| Wenn Zahlen ständig geändert werden, sind die Chancen zu gewinnen geringer, als wenn immer dieselben Zahlen verwendet werden | ☐ |
| Interpretationsverzerrung | |
| Gewinne werden mit Fähigkeiten und Fertigkeiten in Verbindung gebracht | ☐ |
| Verluste werden auf Pech und schlechte Umstände, und/oder Wahrscheinlichkeiten zurückgeführt | ☐ |
| Die Erinnerungen an den letzten Gewinn führt dazu, dass das Glücksspiel fortgesetzt wird | ☐ |
