# Supplementary material for: Automatic detection of problem-gambling signs from online texts using large language models
Source: PLOS Digit Health. 2024 Sep 25;3(9):e0000605. doi: 10.1371/journal.pdig.0000605 (PMC11423982; doi:10.1371/journal.pdig.0000605)
Supplement: S2 File — (PDF) [file pdig.0000605.s002.pdf]

The following post has been annotated as target, since the person appears to display several common gambling-related cognitive distortions, such as the tendency to believe that losing is a result of external factors (such as the game being rigged or manipulated) rather than a result of random chance, the belief that specific numbers related to the chances of winning (illusion of control), and the belief in hot streaks (predictive control):

“Also ich bin jetzt echt schon krass überzeugt, dass Account-gebundenes Spiel bzw Gewinne stattfinden. Hab 5 Euro bei Unibet einbezahlt. Damit BoD gespielt und mit 1 Linie auf 45 Euro geklettert. Eigentlich toll. Dann ab zu Millionaire. Mal auf 0,20 einige Spins laufen lassen, Balance hat sich nicht groß verändert. Dann auf 0,60. Das Spiel bezahlte toll und immer wieder kam ich auf meine 40-50 Euro. Dann kamen auch schon die Freespins. 14 Stück beim Ersten. 14 Freispiele in Millionaire auf 0,60. Ergebnis: Unfassbare e 2,20. Das konnt ich schon fast nicht glauben, aber gut. Einige Spins später, wieder Freespins auf 0,60. Jetzt aber. Diesmal bei 12 Spins gestoppt. Ergebnis diesmal mit 0,60: Noch unfassbarere e 0,54. Also nichtmal X1. Aus 12 Spins. Insgesamt 11 Leerspins aus den 12. Ganz im Ernst jetzt, ich hab schon schlechte Freespins in dem Spiel erlebt, aber das 2x in Folge war ne Premiere. Vor allem so schlecht, dass es wahrscheinlich besser gewesen wär, das Basegame wär weiter gelaufen statt den Freespins. Danach natürlich nichts mehr. Überhaupt nichts. Die 40 Euro liefen durch, alles weg. Bin mal weg hier und auch aus den OC’s. Das is einfach zu heftig und ich glaub jetzt auch echt nicht mehr, dass da auch nur irgendwas zufällig passiert.” [So I’m now really convinced that account-bound play and winnings are happening. I put in 5 euros at Unibet. Used it to play BoD and climbed up to 45 euros with one line. Really great. Then went over to Millionaire. Let some spins run on 0.20, balance didn’t change much. Then on 0.60. The game paid well and I kept getting back to my 40-50 euros. Then came the free spins, 14 at first. 14 free spins in Millionaire on 0.60. Result: Unbelievable e 2.20. I almost couldn’t believe it, but okay. A few spins later, free spins again on 0.60. But this time, stopped after 12 spins. Result this time with 0.60: Even more unbelievable e 0.54. Not even x1. Out of 12 spins. In total, 11 empty spins out of 12. Honestly now, I’ve already had bad free spins in the game, but this 2x in a row was a premiere. Especially so bad that it would probably have been better if the base game had continued instead of the free spins. After that, of course, nothing more. Absolutely nothing. The 40 euros ran through, everything gone. I’ll be gone here and also out of the OCs. It’s just too intense and I really don’t believe anymore that anything happens randomly.]

The following post has been annotated as target, since the person describes unsuccessful efforts to control gambling, financial problems related to gambling, chasing losses, and lying to conceal the extent of involvement with gambling:

“Hallo Ich habe vor ca 4 Monaten angefangen zu spielen .und auch gleich fett gewonnen bei Trada waren es in kürzester Zeit 7000 Euro mit einer Ein- zahlung von 25 Euro. Und so ging es auch im Rizk und Videoslots weiter. Wie das so ist man denkt es geht immer weiter .leider war dem nicht so.ich habe alles wieder verspielt insgesamt über 20000 Euro .genau weiss ich es nicht .ich habe teilweise lastschriften zurückgeholt und lauter so einen Sche- iss. Mein Mann weiss nichts davon .Gott sei dank hab ich die Geschenke unserer Kinder schon gekauft und den monatseinkauf auch.sonnst sähe es böse aus diesen Monat. Das Konto ist komplett leer .Rechnungen sind nicht bezahlt.und das alles nur weil ich mich nicht im Griff hatte . Mein Mann weiss nichts davon .ich denke schon das es besser wäre einfach Schluss zu machen. Ich habe mich jetzt überall selbst gesperrt und hoffe das ich es auch durchhalte .ich weiss nicht wie ich das alles meinem Mann erklären soll . Ich hab immer den Verlusten nachgejagt.ein Riesen Fehler.ich könnt mich selber erschlagen .” [Hello I started playing about 4 months ago and immediately won big at Trada, it was 7000 euros in no time with a deposit of 25 euros. And so it went on in Rizk and Videoslots. As it is, you think it goes on and on. Unfortunately, that wasn’t the case. I gambled it all away again, a total of over 20,000 euros. I don’t know exactly. I partly recalled direct debits and all kinds of shit. My husband doesn’t know anything about it. Thank God I’ve already bought our children’s presents and the monthly shopping. Otherwise it would look bad this month. The account is completely empty. Bills are not paid. And all because I didn’t control myself. My husband doesn’t know anything about it. I think it would be better to just break up. I have blocked myself everywhere and hope I can keep it up. I don’t know how to explain all this to my husband. I’ve always chased the losses. A huge mistake. I could beat myself to death.]

The following post has been labelled as inconclusive. The fact that the person is asking these questions suggests that they may have concerns about their own gambling beha- viour and may be seeking information to determine if they exhibit signs of gambling addiction. However, without further context or information, it is not possible to say whether the person is actually struggling with gambling addiction:

“Ab wann gilt man eurer Meinung nach als spielsüchtig? Kann man spielsüchtig sein, ohne im Minus zu sein? Wie hoch sind eure größten Verluste?”
[At what point do you think someone is considered addicted to gambling? Can one be addicted to gambling without being in the red? What are your largest losses?]

The following post has been annotated as non-target, since it contains a question about the reputation of a casino:

“Hallo Liebe Leute . Kann mir jemand von euch sagen ob das Fairplay Casino seriös ist, und ob es umbedenklich ist dort einzuzahlen ?? Danke euch schon mal im vorraus ! Lg an alle”
[Dear people. Can any of you tell me if Fairplay Casino is reputable and whether it’s safe to deposit there?? Thanks in advance! Regards to all]
